# Sleep, Circadian Health and Melatonin for Mitigating COVID-19 and Optimizing Vaccine Efficacy

**DOI:** 10.3389/fnins.2021.711605

**Published:** 2021-08-18

**Authors:** Rachel U. Lee, Gena L. Glickman

**Affiliations:** ^1^Department of Allergy, Immunology & Immunizations, Walter Reed National Military Medical Center, Bethesda, MD, United States; ^2^Department of Psychiatry and Neuroscience, Uniformed Services University of the Health Sciences, Bethesda, MD, United States

**Keywords:** sleep, circadian rhythms, melatonin, COVID-19, immune system, vaccination

## Introduction

Over the past sixteen months, more than 4 million lives have been lost to COVID-19, with over 600,000 deaths in the United States alone. In addition to the growing death toll and negative impact on physical and mental health, the pandemic has led to a multitude of secondary ramifications stemming from economic and social devastation on an unprecedented scale. Despite tremendous efforts by scientists, healthcare workers, pharmaceutical companies, government agencies and many other entities, we are still far from the return to normalcy that most hold as a goal.

COVID-19 represents a wide spectrum of disease caused by the SARS-CoV-2 virus, including asymptomatic carriage, mild respiratory illness, multi-system inflammatory disease, long-hauler chronic autoimmune-type syndrome, respiratory failure and death. A key clinical feature that coincides with greater disease severity, lingering morbidity, and mortality is an uncontrolled and dysregulated inflammatory response to infection (Blanco-Melo et al., 2020). Although there are still areas of uncertainty about duration of immunity to SARS-CoV-2 and long-term health implications, certain factors have already been identified and associated with milder disease and better prognosis (e.g., younger age, female sex, and healthy weight) (Wolff et al., 2021).

It is clear that vaccination will play a vital role in overcoming this pandemic. Yet, vaccination alone is not a panacea. Some will still get COVID-19 despite vaccination; others have and will become ill before the vaccine becomes available to them; still others will never be vaccinated, either due to misguided fears or contraindications due to particular medical conditions and allergies. In spite of the extraordinarily rapid development and high efficacy of the current vaccines, new variants or other significant mutations may render them obsolete, and unfortunately, currently approved COVID-19 therapies have only modest efficacy and are not without risk and expense (Callaway, 2021). Thus, it is imperative that we seek to mitigate the negative impacts of COVID-19 with every tool in our arsenal.

Many of the risk factors identified to date are *not* modifiable, such as age, genetics, and others; however, there are some relatively untapped and modifiable factors that we should keep in mind in our fight against COVID-19 (Wolff et al., 2021). Sleep, circadian health and melatonin are three different, albeit related, considerations that may serve to enhance the immune system, boost vaccine efficacy, and exploit vulnerabilities of SARS-CoV-2.

## Sleep and Immunity

Sufficient sleep is a known pillar of good physical and mental health. Perhaps most directly relevant to COVID-19, sleep boosts innate and adaptive immune responses, serving the dual role of augmenting the body's ability to fight off infection itself and improve response to vaccine (Irwin, 2019). Well before COVID-19, studies found that regular sleep of sufficient duration and quality strengthens the immune system and enhances response to vaccine (Besedovsky et al., 2019; Irwin, 2019; Haspel et al., 2020). In particular, several studies have demonstrated that increased sleep in the period immediately before or after vaccination confers added protection against influenza as well as hepatitis A and B (Spiegel et al., 2002; Lange et al., 2011; Prather et al., 2012, though see Benedict et al., 2012 for possible sex differences). While such studies were carried out in the context of other viruses and vaccines, there is no reason to think that COVID-19 is any different.

**Figure 1:**
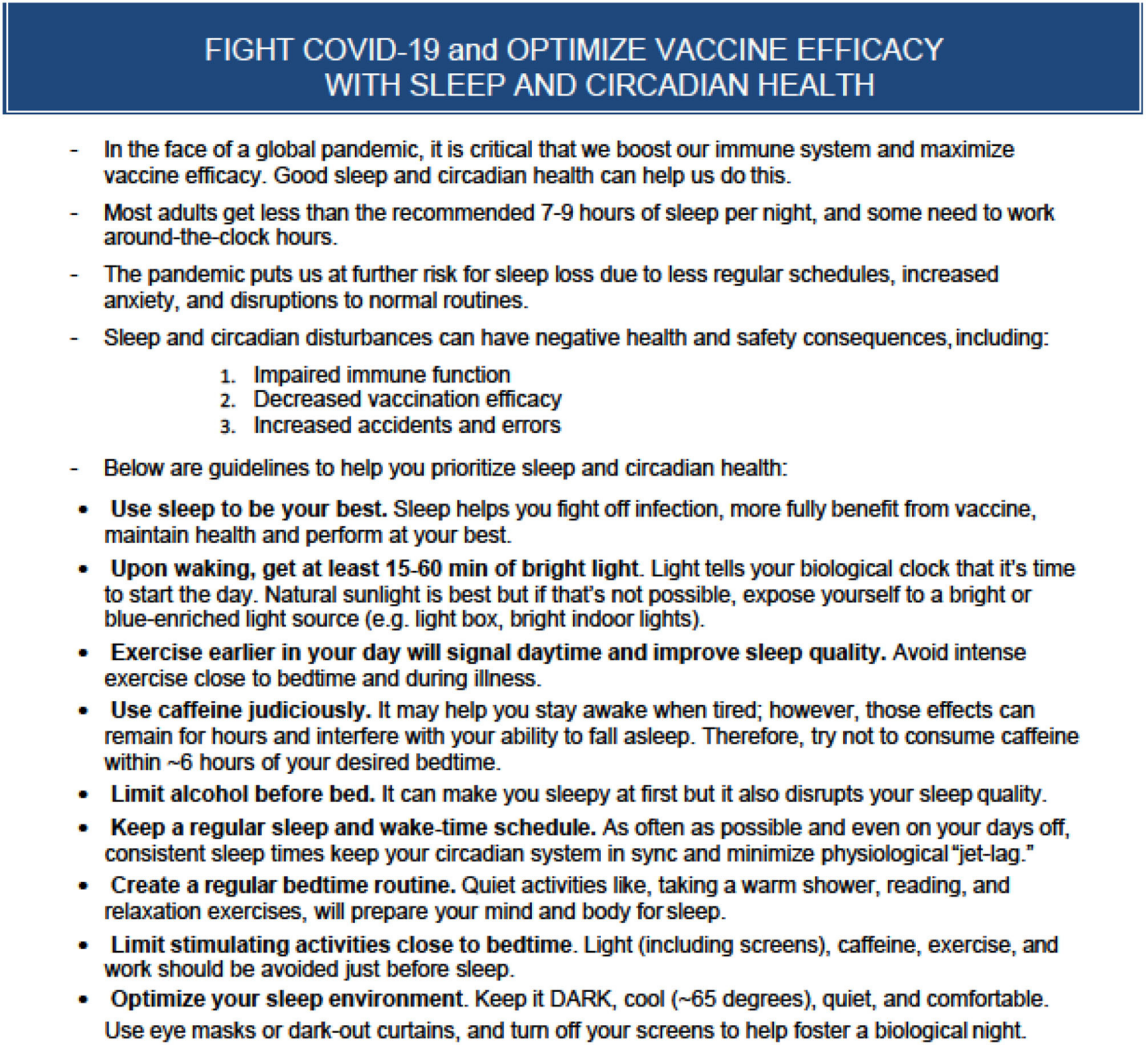
Fight COVID-19 and optimize vaccine efficacy with sleep and circadian health. A summary of the role of sleep and circadian health in immune system function along with concrete tips for supporting sleep and circadian health.

Future research will further elucidate the relationship between sleep and COVID-19 more specifically, but the pandemic puts us at increased risk for sleep loss right now. In addition to illness, other consequences of the pandemic, such as increased stress and anxiety, disruptions to normal routines, less regular work and school schedules, and more time on screens, may all result in sleep disturbances, giving the virus another leg up on us. It is important that we recognize and address the significant problem this poses by reminding the public of the importance of sleep in optimizing health and offering concrete steps for maximizing sleep quality and quantity during these unusual times (see Figure 1).

## Circadian Health and Immune Function

One of the primary regulators of sleep is the circadian timing system, which is responsible for the temporal coordination of a host of physiological processes, including not only sleep but immune system function as well (Besedovsky et al., 2019; Haspel et al., 2020). Under normal healthy conditions, both the amount and reactivity of circulating immune cells predictably fluctuate across the day and night (Haspel et al., 2020). It is therefore not surprising that myriad studies of circadian clock disruption demonstrate a corresponding increase in disease severity across a variety of physical and mental health conditions (Haspel et al., 2020). Circadian disruption, as commonly occurs with jet lag or shiftwork, not only impairs sleep and immune response (and thus, indirectly increases risk of infection) but can also cause a hyper-inflammatory state on its own, which may increase COVID-19 symptoms and severity (Besedovsky et al., 2019; Haspel et al., 2020). In addition, shiftwork has been associated with reduced humoral response to meningococcal vaccination (Ruiz et al., 2020), and this may generalize to other vaccines, such as those recently developed for COVID-19. Taken together, this is of particular concern for all frontline essential workers (i.e., hospital staff, police, fire, etc.) who are on non-standard schedules due to the need to provide around-the-clock care or services, as circadian disturbance coupled with exposure to the virus and/or reduced vaccine efficacy may compound their risk for infection and more severe disease.

Circadian clocks in most tissues and organisms affect immune response to vaccination; as such, the time of day when the vaccine is administered is also important. Morning vaccination has been shown to induce a more robust adaptive response as compared to evening administration in model systems (Nobis et al., 2019) and with the antituberculosis bacillus Calmette-Guérin (BCG) vaccine in humans (De Bree et al., 2020). Viral replication is also under circadian control, and though it has not been reported to our knowledge, SARS-CoV-2 almost certainly interacts with the host circadian system. Thus, if the severity of COVID-19 symptoms and clinical outcomes are the result of a battle between viral replication and host immune response, enhancing circadian health in humans and capitalizing on temporal vulnerabilities of the virus via deliberately timed vaccination and treatment may also allow us to boost their efficacy and further reduce the severity of symptoms in those with COVID-19 (Besedovsky et al., 2019; Haspel et al., 2020; Schneider et al., 2020).

## Melatonin's Role in Sleep and Immune Health

Melatonin is a hormone produced and secreted by the pineal gland that is regulated by, and feeds back on, the circadian clock. In healthy people on regular schedules, melatonin is high at night and low during the day. In addition to daily fluctuations, overall levels of the hormone change across the lifespan, peaking in early childhood and decreasing significantly with age (Schneider et al., 2020). Considering the hormone's anti-inflammatory and immune-enhancing properties, greater protection from COVID-19 due to higher melatonin levels is one putative explanation for why children are largely spared from the most severe cases of COVID-19 (Schneider et al., 2020). Exogenous melatonin is a commonly prescribed treatment for patients with sleep and circadian disorders. While it is readily available as an over-the-counter supplement in the United States, there is considerable variability in the quality of those products, which are not FDA-regulated. That said, melatonin has been found to be unusually safe in humans when taken in supplement form (Schneider et al., 2020).

In addition to its role in supporting sleep and circadian health, melatonin's pleotropic effects include anti-inflammatory, immune regulatory and antioxidant benefits. Consequently, treatment with melatonin may improve immune system function, provide protection from damage to cells under inflammatory conditions, and prevent pathogenic inflammation that may lead to autoimmunity (Besedovsky et al., 2019; Haspel et al., 2020; Schneider et al., 2020). Melatonin reduces pro-inflammatory cytokines, such as TNF-a, IL-1b, IL-6, IL-8 and IL-17 while increasing levels of anti-inflammatory cytokines (Cardinali et al., 2020). The hormone further influences immune response via stimulating the proliferation and maturation of various specialized cells (e.g., natural killer, T and B lymphocytes, granulocytes, and monocytes) that enhance adaptive immunity (Miller et al., 2006). Consistent with those findings, early studies show potential utilty of melatonin as a vaccine adjuvant (Cardinali et al., 2020). Finally, melatonin also has important antioxidant properties, which help prevent cellular damage and attenuate the tissue-damaging effects of inflammation, translating to treatment efficacy in patients with sepsis, diabetes, cardiomyopathy, and multiple sclerosis, all comorbidities that relate to COVID-19 risk (Cardinali et al., 2020).

## Conclusion

With all the challenges surrounding public health measures, healthcare equity and vaccination distribution, just to name a few, there is a clear need for strategies that everyone can do *now* to maximize their health and minimize the impact of the pandemic. Sufficient sleep, optimal circadian health and exogenous melatonin may offer a potent trifecta countermeasure. These three factors not only serve to reinforce one another but each has been shown to play a critical role in immune response to infection, immune modulation in preventing unchecked inflammation and autoimmunity, and overall health. Providing the public with guidance on how to optimize sleep and circadian health, and studying inexpensive and accessible supplements such as melatonin, may reduce potential SARS CoV-2 infection and help promote vaccination efficacy.

## Author Contributions

RL and GG: conceptualization and writing. GG: funding acquisition. Both authors contributed to the article and approved the submitted version.

## Author Disclaimer

The authors are a United States military service member and employee of the U.S. Government. This work was prepared as part of their official duties. Title 17 U.S.C.105 provides that ‘Copyright protection under this title is not available for any work of the United States Government.’ The views expressed in this article are those of the authors and do not reflect the official views or position of the Department of the Army/Navy/Air Force, Department of Defense, or U.S. Government.

## Conflict of Interest

GG reports that she has received equipment, advice, or financial support and/or served as a consultant to Philips, Litebook, BIOS Lighting, f-lux, PennWell Corporation, LightShow West, and Well Building Institute and that she holds four currently issued patents (USPTO 7,678,140, 8,366,755, 10,213,619, and 10,603,507) and two continuing patent applications (USPTO 16/657,927 and 16/831,737). She also has a sleep education program under development with colleagues, entitled Circadian, Light and Sleep Skills, from which much of the information in the figure was derived. The remaining author declares that the research was conducted in the absence of any commercial or financial relationships that could be construed as a potential conflict of interest.

## Publisher's Note

All claims expressed in this article are solely those of the authors and do not necessarily represent those of their affiliated organizations, or those of the publisher, the editors and the reviewers. Any product that may be evaluated in this article, or claim that may be made by its manufacturer, is not guaranteed or endorsed by the publisher.
